# Size-dependent tradeoffs in seasonal freshwater environments facilitate differential salmonid migration

**DOI:** 10.1186/s40462-019-0185-1

**Published:** 2019-12-21

**Authors:** Philip Dermond, Carlos J. Melián, Jakob Brodersen

**Affiliations:** 1 Department of Fish Ecology and Evolution, EAWAG Swiss Federal Institute of Aquatic Science and Technology, Centre of Ecology, Evolution and Biogeochemistry, Seestrasse 79, CH-6047 Kastanienbaum, Switzerland; 2 Institute of Ecology and Evolution, Aquatic Ecology, University of Bern, Baltzerstrasse 6, CH-3012 Bern, Switzerland

**Keywords:** Differential migration, Predation, Growth, Tradeoffs, Salmonid, Freshwater

## Abstract

**Background:**

Seasonal spatio-temporal variation in habitat quality and abiotic conditions leads to animals migrating between different environments around the world. Whereas mean population timing of migration is often fairly well understood, explanations for variation in migratory timing within populations are often lacking. Condition-dependent tradeoffs may be an understudied mechanism that can explain this differential migration. While fixed condition-specific thresholds have been identified in earlier work on ontogenetic niche shifts, they are rare in differential migration, suggesting that thresholds in such systems can shift based on temporally variable environmental conditions.

**Methods:**

We introduced a model based on size-specific tradeoffs between migration and growth in seasonal environments. We focused on optimal migratory timing for first-time migrants with no knowledge of an alternative habitat, which is a crucial stage in the life history of migratory salmonids. We predicted that optimal timing would occur when individuals move from their natal habitats based on a seasonally variable ratio of predation and growth. When the ratio becomes slightly more favorable in the alternative habitat, migratory movement can occur. As it keeps shifting throughout the season, the threshold for migration is variable, allowing smaller individuals to move at later dates. We compared our model predictions to empirical data on 3 years of migratory movement of more than 800 juvenile trout of varying size from natal to feeding habitat.

**Results:**

Both our model and empirical data showed that large individuals, which are assumed to have a lower predation risk in the migratory habitat, move earlier in the season than smaller individuals, whose predicted predation-to-growth ratio shifted to being favorable only later in the migratory season. Our model also predicted that the observed difference in migratory timing between large and small migrants occurred most often at low values of growth differential between the two habitats, suggesting that it was not merely high growth potential but rather the tradeoff between predation and growth that shaped differential migration patterns.

**Conclusions:**

We showed the importance of considering condition-specific tradeoffs for understanding temporal population dynamics in spatially structured landscapes. Rather than assuming a fixed threshold, which appears to be absent based on previous work on salmonids, we showed that the body-size threshold for migration changed temporally throughout the season. This allowed increasingly smaller individuals to migrate when growth conditions peaked in the migratory habitat. Our model illuminates an understudied aspect of predation as part of a condition-dependent tradeoff that shapes migratory patterns, and our empirical data back patterns predicted by this model.

## Background

Migration is a movement tactic undertaken by many different taxa across the globe. These fascinating movements can represent the movement of a large proportion of populations and biomass between ecosystems. A key component to migratory movements is timing. As migrant individuals are often tracking temporary resource peaks [1, 2], early or late initiation of migratory movement can have direct consequences for fitness, resulting in missed opportunities for growth or even death [3].

Differential migration, i.e. the variation among individuals from the same population in regards to timing and/or destination of their migration, has been widely studied [4, 5]. Many factors affecting migratory timing have been identified [6]. Optimal migration theory suggests that timing is not determined solely by external, environmental factors, but also by individual traits [7], and individuals in most populations do not migrate at the same time even when tracking the same type of resources [5, 8]. Individual traits such as body size can influence physiological abilities or other factors like predation risk, thereby changing the optimal time for departure [9, 10]. Despite a large body of empirical and theoretical work identifying influential factors, conclusive mechanistic explanations for the occurrence of differential migration are lacking. As competing hypotheses can often make the same prediction for migratory timing based on the same factors (e.g., [11]), the complexity of migratory behavior makes the influence of environmental factors difficult to disentangle. While previous work on habitat shifts has suggested fixed thresholds at which movement should occur in some systems [12], such thresholds seem absent in differential migration [13, 14].

Many aspects of animal life and behavior are governed by condition-dependent tradeoffs. Examples are found in diverse fields across biological sciences, including a multitude of examples in evolution (e.g., [15–18]) and ecology [19–22]. While tradeoffs can sometimes be circumvented when fitness consequences are severe, they are often unavoidable [23]. Acquisition tradeoff theory (e.g., [24]) suggests that a strategy with large benefits such as high resource acquisition and higher growth can be associated with a corresponding cost, such as increased mortality through higher predation [25, 26]. Such tradeoffs between mortality and growth (i.e., μ/G) have been shown to influence not only time spent foraging, but also ontogenetic habitat shifts [27]. Once a partial size refuge from predation has been reached, it is possible to move from a low-risk, low-benefit to a high-resource, high-predation environment [27].

Despite ample evidence that state-specific tradeoffs, involving traits such as size, condition, or others, can influence seasonal movement and potentially dispersal, migration research has thus far not focused much on the importance of tradeoffs in determining variance in patterns of differential migration (but note [28] for exceptions, [29, 30]). Instead, physiological factors or barriers are often considered to be of higher importance [31–33], even though they are not always supported by empirical data (e.g., [34, 35]). However, tradeoffs between the size- and seasonal-specific changes of growth and predation risk may also affect the optimal time of migration for individuals of different body sizes. As predators are generally limited in their abilities to consume prey that exceed a certain fraction of their own body size for various reasons [36], larger prey individuals face lower specific risks in highly productive and predator-rich environments. However, as conditions change temporally and feeding environments increase in productivity, smaller individuals are able to move to more productive environments as their growth potential outweighs predation risk [13] and leads to a temporally shifting rather than fixed size threshold. An influence of size on timing has been shown in previous studies of salmonid migration and the possibility of predation as an explanatory factor has been proposed [37–39], but the tradeoff between growth and predation has to our knowledge never been robustly tested as a main cause, despite the ideal suitability of salmonid migration to address such questions. Proximate cues such as discharge or temperature have been studied thoroughly in salmonids [40–42]. However, unlike in other systems where first-time migrants can learn from parents or conspecifics [43–45], the majority of juvenile salmonids are first-time migrants that move into an unknown environment at a time when their parents and older conspecifics have long left the natal stream [46, 47]. State-specific tradeoffs present ultimate causes that can select for optimal timing and should therefore be considered when studying mechanistic explanations for differential migration. Nevertheless, the existence and magnitude of effect of state-specific tradeoffs remain understudied in general [48, 49].

Salmonid migration is remarkable in both its extent and diversity. Individuals from the vast majority of salmonid species migrate from a natal to a feeding environment before returning for reproduction [50]. The feeding environment generally offers higher growth rates, with migratory salmonids reaching much larger sizes at younger ages than resident conspecifics [51–54]. Despite fitness-related benefits potentially associated with early arrival (*sensu* the “early bird” hypothesis, [55]), the time spent in natal environments and seasonal timing of migration vary greatly between species and even populations [56–59]. Body size has been shown to strongly influence migratory timing, with larger individuals generally migrating earlier [60–62]. With habitat-specific growth and predation risk both being affected by size, a growth-mortality tradeoff could be influencing the timing of migration [26, 27]. Indeed, migration in salmonids is a strategy that offers high growth opportunities, but also carries a high risk of mortality [54, 63–65]. Work on migratory salmonids is generally undertaken on anadromous populations, i.e. those moving between freshwater and saltwater. Studying the influence of predation as a driver of migratory timing has therefore been difficult due to a physiological barrier that is both size- and temperature-dependent, likely shaping migratory timing [66, 67] and leading to predictions similar as those of predation to growth tradeoffs. To remove a potentially confounding effect of such a barrier, freshwater migration systems that do not require a change of osmoregulation can be used.

In this study, we use theory to explore various parameters and their influence on the timing of migration and test our model with empirical data. We introduce a conceptual model based on a risk-reward tradeoff between predation and growth. This model describes the conditions under which an individual of a given size will migrate from its natal to its migratory or feeding habitat, accounting for the temporal development of the ratio of size-specific predation pressure to temperature and size-dependent growth. This simple model estimates a seasonally shifting size threshold for migration, predicting that individuals should move from one environment to the other when the ratio of predation risk and potential growth shifts towards growth, irrespective of the actual values for these parameters. We compare our results to 3 years of empirical data on the migration of several hundred Atlantic trout (*Salmo trutta*) from twelve tributary streams to a large Swiss pre-alpine lake, Lake Lucerne. We hypothesize that migratory patterns of first-time migrants in this study system can be predicted based solely on a risk-reward tradeoff, with no physiological barriers, and that larger fish will move out of streams earlier during freshwater migration. If our model and empirical data bear out these hypotheses, we suggest freshwater migration ecology should also consider biotic interactions such as predation or competition along with classic physiological barriers such as temperature or salinity, i.e. environmental factors.

## Material and methods

### Study system

**Fig. 1:**
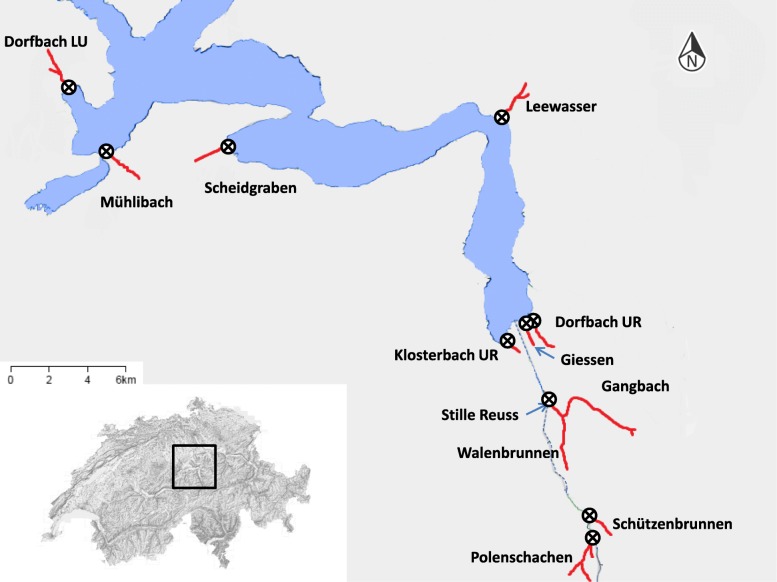
Map of study sites, with study streams in red and map of Switzerland for reference (modified from map.geo.admin.ch). Crossed circles indicate the positions of PIT antennas

We investigated timing of Atlantic juvenile trout (*S. trutta*) migration from twelve tributaries into Lake Lucerne in central Switzerland (Fig. 1, Additional file 1: Table S1). Lake Lucerne is a large (113.72 km^2^, max depth 214 m), pre-alpine lake that is dominated by perch (*Perca fluviatilis*) in the littoral zone and whitefish (*Coregonus spp.*) in the pelagic zone. The piscivorous community is dominated by size-structured populations of large-bodied perch and pike (*Esox lucius*). The latter attain sizes exceeding 100 cm in length, subjecting all juvenile trout to some degree of predation risk. Lake-migratory trout can also reach sizes of over 90 cm, with most spawning trout ranging between 40 and 50 cm in length. Growth-rate as documented through recaptures of individuals of both movement tactics is significantly higher for lake-migratory than for stream-resident trout (Additional file 1: Table S2).

A large number of trout-dominated streams with highly variable temperature and flow regimes feed this lake, and extensive adfluvial migrations are known in the system [68]. Earlier work has shown trout populations in some of these streams to be at least partially reproductively isolated, with significant fixation indices (F_ST_s) between many of them [69], likely due to natal-stream homing of migrants and philopatry of resident individuals.

### Model

We built an a priori conceptual model to investigate first-time migratory timing from natal to feeding habitat. This model predicted differential size-specific timing of migration in a seasonally changing environment by accounting for the ratio of size-specific predation pressure to growth, which is also temperature and size-dependent. As we worked with juvenile trout, we drew on a model by Elliot et al. [70] to model growth based on temperature and hypothetical resource availability, which we assumed to be higher in the lake. Temperature was modeled to follow typical seasonal patterns for the temperate zone, and specific growth was set to be higher for smaller fish, which is a well-documented general pattern in trout [70]. We partitioned size-specific predation pressure present in the lake, *L*, and in the stream, *S*, in two components: *α*_*L*_ (*α*_*S*_), and *P*_*Lmax*_ (*P*_*Smax*_), where *α*_*L*_ *(α*_*S*_*)* is the percentage of predators capable of preying on an individual of a certain size, M, and *P*_*Lmax*_ (*P*_*Smax*_) is the predator carrying capacity in the lake (stream). We were thereby able to model a difference in predation pressure between habitats by setting a higher *P*_*max*_ in the lake, and model gape limitation of piscivorous predators by decreasing *α* for larger fish. Our model described the condition for an individual of size *M* to migrate from stream *S* to lake *L* as

1 $$ \left(\frac{P_L(M)}{G_L(M)}\right)\le \left(\frac{P_S(M)}{G_S(M)}\right) $$

where *P*_*L*_*(M)* and *P*_*S*_*(M)* represent predation pressure of lake *L* (stream *S*) for an individual of size *M*, and *G*_*L*_*(M)* and *G*_*S*_*(M)* represent temperature dependent growth, modeled based on equations from Elliot et al. [70], in lake *L* (stream *S*) for an individual of size *M*. To explore the influence of tradeoffs between predation and growth in the stream and the lake on optimal migratory timing, we also considered differential growth between the lake and the stream for an individual of size *M*, Δ*G(M)*, as

2 $$ \left(\frac{P_L(M)}{\varDelta G(M){G}_L(M)}\right)\le \left(\frac{P_S(M)}{G_S(M)}\right), $$

Δ*G(M)* is a multiplier representing differential growth between habitats, with large values indicating faster growth in the lake compared to the stream (e.g., at a value of Δ*G(M)* = 3 the potential for growth is three times as high in the migratory habitat as in the natal habitat). This means that in the migratory habitat, a high ΔG(M) leads to higher growth for individuals of a given size at a given temperature. Based on this simple model we were able to predict the time of migration between individuals of different size to contrast the generality of our predictions with the reported empirical patterns.

After collecting and analyzing the empirical data, we performed an *a posteriori* qualitative comparison of the model predictions and observed migratory patterns. We additionally used iterative simulation modeling to explore parameter values for the percentage of predators capable of preying on large individuals of size M’ in the lake and the stream that were randomly sampled from a uniform distribution with ranges α_L = [1, 5] and α_S = [0.01,3], respectively. For small individuals, M, the ranges were randomly sampled from α_L = [1, 5] and α_S = [0.1,1], respectively. Finally, we used simulation modeling to study the full space of growth, predation, and growth differential in the two habitats to predict which values would be most likely to create differential migration based on body size.

All modeling was conducted in GNU Octave, version 4.2.2 [71].

### Capture of juvenile trout

We collected juvenile trout from 12 streams (Fig. 1) during February and March of 2015, 2016 and 2017. These dates cover the time shortly before migration starts in our system. We used a DC backpack electrofishing device to capture trout by moving along a stretch in the upstream direction and removing trout large enough for tagging. We electrofished each stream at least twice during the sampling period and targeted different sections on each visit. We thereby included fish from at least four sections of each stream, spread along most of the stream’s length. This was done to ensure that the migratory patterns we observed were representative of the whole population.

### Tagging

We tagged a total of 3812 trout (see Additional file 1: Table S2 for trout by stream), with a total length ranging between 104 and 250 mm in twelve streams, using 23 mm 0.6 g half-duplex passive integrated transponder (PIT) tags from Oregon RFID (Oregon, USA). Fish were tagged close to the migration period to ensure that both mortality and growth between tagging and migration was minimal. The trout tagged were all juveniles that had not yet reproduced or performed their first migration to the feeding environment. The minimum total length for tagging was based on the recommendations from Larsen et al [72], with an added size margin for safety. We anesthetized fish by immersing them in a bath of MS-222 concentrated at 0.067gl^− 1^ until they did not respond to touch. We then measured total length of the fish to the nearest mm and weighed them to the nearest 0.1 g (mean ± SD: TL = 160.7 ± 55.7 mm, weight = 58.9 ± 132.5) before surgically implanting the tags. We used a scalpel to make a small incision and placed the tag into the body cavity of the fish and then treated them with Koi Med Wound Snow©. We elected not to close the wound with staples or stitches, as studies suggest that this increases the risk of infection [73] without significantly improving survival of the tagged fish or tag retention. After tagging, we allowed fish to recover in well-oxygenated water and then immediately returned them to the section they were caught from. As our model could potentially predict migratory behavior for individuals that did not reach maturity in their first summer in the alternative habitat, additional electrofishing surveys were carried out in October, November and December of 2015 and 2016, as well as November and December of 2017, to assess the size and maturity of returning migratory trout. For these, we recorded measurements of length and weight and assessed whether sexual maturity had been reached. The study was carried out with permission from fisheries authorities and regulatory bodies for animal experimentation. All methods and the handling of live fish were assessed by the regional veterinary office regulating animal experimentation and approved under permit numbers LU01/14 and LU08/17.

### Migration monitoring

All sampled streams were equipped with a dual loop-antenna system connected to a multiplex PIT-tag reader (Oregon RFID), allowing us to record both timing and direction of migration. One antenna monitors multiple populations due to the confluence of streams occurring above the antenna location (Walenbrunnen and Gangbach are monitored by the antenna in Stille Reuss, Fig. 1). While most antennas were directly located at the outflow into the lake, some antennas were placed further upstream at the confluence with a major tributary before the stream widens and deepens too much for an antenna to be placed. As the fish passing these antennas have to enter either a major tributary or a deep section of river with low fish density close to the lake, we considered them as migrants if they were last recorded on the downstream antenna. We then used this last record as time of migration.

### Data analysis

We used linear models to assess the effect of size on migration date (lm date~size), and controlled for both the river of origin(lm date~size+river) and the year of migration (lm date~size+river+year). We used streams as replicates and analyzed within-stream differential migration, which allowed us to control for proximate cues such as discharge or photoperiod while focusing on ultimate effects controlling migratory timing. Statistical analysis was performed using packages “base” and “stats” in R version 3.5.2 [74].

## Results

### Predicted size-dependent timing of migration

**Fig. 2:**
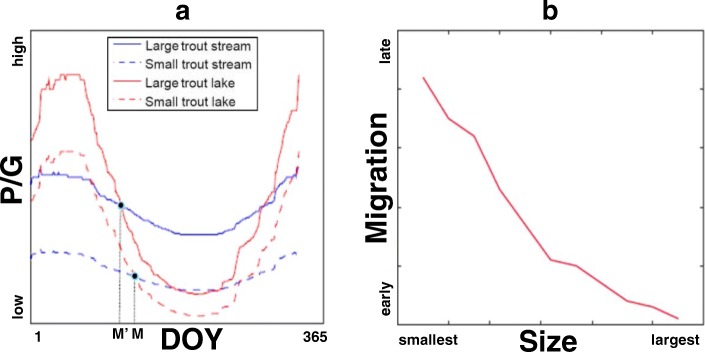
**a** Development of the ratio between predation and growth (y-axis, moving from low to high) for large (M’, solid line) and small (M, discontinuous line) fish over time (Julian DOY on x-axis) in the natal (blue stream) and migratory habitat (red, lake). Low values of P/G show a high growth potential compared to the predation risk, with the growth in the denominator outweighing predation in the numerator. Crossing points between red (lake) and blue (stream) lines indicate the optimal time of migration based on a differential growth, ΔG(M), equal to 5 and higher predation risk for smaller individuals in the lake. **b** Optimal time of migration (ranging from early to late in the migratory season on the y-axis) from stream to the lake for fish of different sizes (ranging from smallest to largest on the x-axis), predicted based on size-dependent-predation and growth in both alternative habitats

Figure 2a illustrates size-dependent differential migration by showing the expected crossing points based on eq. (2) as a function of hypothetical large, *M’*, and small, *M*, individuals drawn from the upper and lower extremes of the modeled size-spectrum. Large individuals will leave sooner than the small individuals for a broad set of combinations of *P*_*L*_*(M)*, *P*_*S*_*(M)*, *G*_*L*_*(M)*, and *G*_*S*_*(M)*, where all crossing points satisfy the inequality condition to leave from the stream, *S*, to lake, *L*. When modeling size as a continuous variable, there is an inverse relationship between size and date of migration, i.e. the largest individuals migrate earliest, with progressively smaller individuals migrating as time passes (Fig. 2b).

Differential growth plays an important role in predicting migration timing. The larger the differential growth between lake, *L*, and stream, *S*, for larger individuals (i.e., Δ*G(M’) > > ΔG(M)*) the sooner individuals of size *M’* will leave the stream with respect to individuals of size *M*. This can be seen following the different ratios from the left term of eq. (2) as

3 $$ \left(\frac{P_L\left(M^{\prime}\right)}{\varDelta G\left(M^{\prime}\right){G}_L\left(M^{\prime}\right)}\right)\le \left(\frac{P_L(M)}{\varDelta G(M){G}_L(M)}\right) $$

Larger individuals have higher predation risk in the lake than the stream, yet the larger growth of the lake environment can compensate for the predation-growth tradeoff earlier than in smaller individuals, which have higher predation risk overall. This decreases the ratio on the left side of inequality 2 triggering earlier migration timing for larger individuals that satisfy condition 3 in the above equation, i.e. the P/G ratio in the lake is lower for larger individuals.

**Fig. 3:**
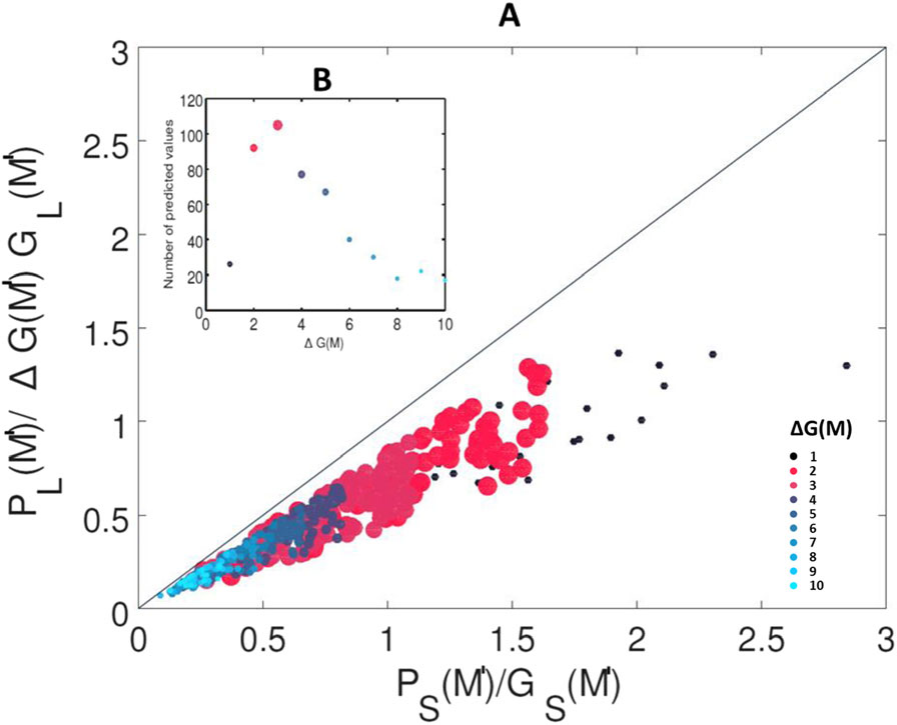
**a** Crossing points where the difference in migratory timing between larger and smaller individuals is within the empirical range of 20 ± 10 days observed in our study system. Colors represent different values of differential growth ΔG(M) (increasing from black to red, magenta, dark blue and light blue dots). The ratio for predation over growth for the migratory environment is on the y-axis and the ratio for the natal environment on the x-axis. For both axes, the ratio is lowest at the intersection of the axes, meaning no predation risk with positive growth potential, and increases when moving away from this intersection. This means that an increase in predation risk is not matched by an increase in growth potential. For the y-axis, a growth differential of ΔG(M’) is applied to model growth differences between natal and migratory habitat and specific growth for a fish of a given size is calculated using the same formula based on size-specific growth rate for both environments. The black line represents the theoretical 1:1 relationship where P/G is equal for both environments. The temporal differences between large and small migrants occur most often at low values of growth differential between the two habitats (red dots). This result suggests that it is not merely high growth potential but rather the tradeoff between predation and growth that shapes differential migration patterns. **b** The number of runs in our simulation that predict earlier crossing points for given ΔG(M’) values. At low values of ΔG(M’), the predation in the migratory environment outweighs the growth potential for most fish regardless of size, leading to a lower number of predicted values. Differential migration within the empirical range happens most often at intermediate values, then decreases again as the migratory environment becomes profitable enough for small fish to risk predation because of the high growth potential, allowing them to move as early as large fish.

The inequality in eq. 2 produces different clusters of crossing points for varying differential growth values (Fig. 3a). Values of differential growth around three (i.e., three times higher in the lake than the stream) produce the highest number of runs that result in differential migration that best match the empirically observed pattern in the study system (Fig. 3b). Other values of differential growth between the habitats are much less likely to produce differential migration in our simulation model. With low growth differentials, the advantage of large individuals gain is minimal, so that earlier migration for these individuals only occurs in rare cases. For differential growth values larger than three, earlier migration of larger individuals can still occur but decays until minimum values as growth opportunities start to outweigh predation pressure even for small fish, allowing them to migrate earlier and match the timing of larger individuals.

### Empirically observed timing of migration

Over 3 years, we registered a total of 824 outmigrating individuals from twelve streams. The migratory period for all years covered 142 days. Migrants moved from the stream to the lake between day of year 40 and 182 (corresponding to February 9th and July 1st in 2015), with a median of 114 (April 23rd). Peak migration was during the months of April and May, with a total of 670 fish (81.6% of all migrants) moving in these 2 months.

**Fig. 4:**
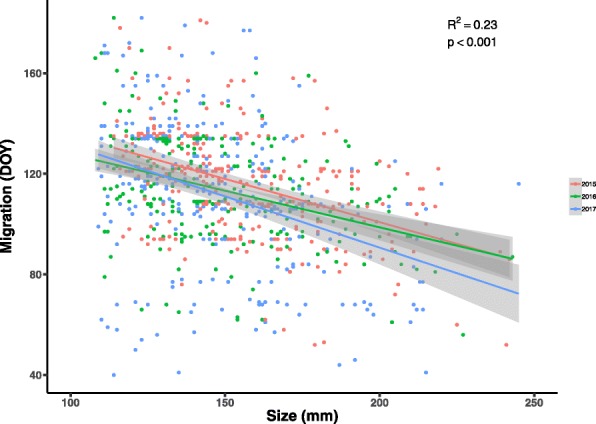
Outmigration date in Julian calendar days as a function of total length. The graph shows 824 individuals that migrated during spring in three separate years, colored by year of migration. The trend is the same for all 3 years, with a significant effect in individual years and combined data for all three

We found a significant negative effect of size on the day of migration, i.e. larger individuals moving earlier than small ones. The best fit came from using stream identity and study year as covariates (date~size+river+year, r^2^ = 0.20, *p* < 0.001, Fig. 4) for the combined data. Controlling for stream identity increases both r^2^ and *p*-values, and the effect is also consistent between years. The difference in timing between the smallest 5% of all individuals (average TL = 112.3 mm) and the largest 5% (average TL = 216.4 mm) is 29 days (largest mean doy = 91 ± 21 SD, smallest mean doy = 120 ± 33 SD). The effect of size on migration is negative for all streams, i.e. large fish migrate earlier in all populations sampled (r^2^-values for individual rivers: mean = − 0.26 ± 0.12).

While our study focuses on juvenile outmigration and not adult spawning migration, our electrofishing data does indicate that a number of juvenile fish that migrate to the lake in spring return to streams in winter before reaching sexual maturity. These fish appear to be overwintering in their natal habitat without reproducing, suggesting that they are performing a non-spawning return migration (Additional file 1: Table S3). This observation matches the predictions of our model for fish that do not reach a size refuge in their first summer in the feeding habitat (Fig. 2a).

## Discussion

The realized migratory behavior of an individual is likely governed by a multitude of biotic and abiotic tradeoffs. In this study, we modeled the onset of trout migration in relation to body size and temperature-dependent growth, which is a key determinant of fitness and survival in this species [75]. First, we developed a model that predicts an earlier onset of migration for larger individuals, based on a predation-growth tradeoff and differential growth between habitats producing a seasonally shifting threshold that allows smaller and smaller individuals to move as growth conditions in the feeding habitat improve. Secondly, we also tested our model predictions with an empirical data set that spans twelve different populations over a three-year period. These data show a significant link between body size and the timing of outmigration which is consistent across years and populations. Migrants do not wait to reach a given threshold, but rather move at a range of sizes throughout the migration period. The largest juveniles migrate from stream to lake roughly 1 month earlier than the smallest, thereby supporting the hypothesis that lower predation risk in larger individuals allows them to move to a riskier, high-growth environment earlier.

The use of generally applicable inequalities in our modeling framework allowed us to iteratively simulate the timing of migration under a range of parameter variations (for both growth and migration) in both a natal and a migratory habitat. The model output remained robust under these varying parameters and consistently predicted differential migration. However, simply increasing the parameter value of differential growth between habitats in our model did not linearly increase the predicted difference in migratory timing between large and small individuals. Rather, earlier migration of large individuals such as we observed in our study system was most likely given a specific combination of growth and predation values, clearly pointing to a tradeoff between these two factors underlying the observed migration patterns. The observed effect of body size on the timing of migration in wild populations among three different years was as consistent as our modeling results, despite yearly variation in the flow and temperature regimes of our twelve study streams. While the average date of migration may temporally change under annually fluctuating environmental conditions, we showed that the effect of body size on migration date remains consistent within populations. Despite the consistent effects, it is important to note that R^2^-values for our linear model are not very high. However, this is to be expected to a degree, as true optimality is likely to be rare in many systems [76]. It is also possible that proximate cues, such as daily runoff or temperature changes, could be influencing migratory patterns on a day-to-day basis (e.g., [77]). Nevertheless, future studies could investigate other potential mechanisms shaping differential migration in freshwater systems.

Body size may be an important predictor of migration timing in various systems for a number of reasons [31, 78, 79]. Ontogenetic habitat shifts or migration to alternate environments should occur when the cost-to-benefit ratio of the alternate habitat is higher than the habitat individuals currently occupy [12], but predation risk is not equal for all individuals. Unfortunately, we were unable to directly measure predation risk of different size classes in the field due to the size and environment of our study system, necessitating some caution in the interpretation of our results. However, the impact of prey size on a predator’s ability to feed on it is well documented [80–83]. Especially in fish, body size can be an important factor in avoiding predation due to the gape limitation of piscivores [84, 85], and it appears plausible that this decreased vulnerability with larger size is also common among other taxa [36]. Other authors have suggested that due to a strong link between growth rates and body size, energetic demand in fast-growing individuals may be more important than actual size, forcing them to move earlier and at smaller sizes due to their higher metabolic rates [86]. While this may be true between seasons or populations, neither our model analyses nor our empirical data support this metabolic hypothesis for within-population differential migration within a single season.

While condition-dependent tradeoffs have been proposed to be underlying mechanistic determinants of migratory decisions in other systems [87], such tradeoffs remain understudied in differential migration. Some work shows migratory birds with low energy stores may prioritize energy acquisition at the expense of predation [88] or migrate earlier to arrive before more competitive conspecifics [89], but such studies on condition-dependent tradeoffs are often confounded by competing hypotheses. Larger body size may predict earlier migration or overwintering closer to breeding sites on account of dominance, higher cold tolerance, and additional factors [49, 90]. In salmonids, larger size may also help with osmoregulation or long migratory journeys, but neither of these factors is sufficiently influenced in our study system. Since we do not have to contend with competing hypotheses, our results present a condition-dependent tradeoff that could be influential enough to stand alone as the reason for differential migratory timing. This raises the possibility that such tradeoffs have been undervalued in systems where physiological barriers are hypothesized to be the main drivers of differential migration.

Various factors that can influence migratory timing have been identified in previous work [78, 91]. Among these are the energy reserves required for migration, which may be especially important in terrestrial movement and if migratory costs are dependent on an interaction between size and time [92]. However, the migration distances in our study system (stream to lake) are generally in the range of meters to no more than a few kilometers, and so both downstream and upstream migration can be achieved with minimal energy investment. Since the general costs and stress of migratory travel (e.g., [93, 94]) are low in our study system, it is possible that this could alter migration tradeoffs in Atlantic trout in favor of residing in the more benign, predator-poor stream environment over winter, when growth is typically limited in both environments due to low temperatures. Interestingly, our model does predict that under some circumstances, certain fish that had only spent a short time in the lake and not yet grown much should overwinter in streams rather than in the lake. In anadromous populations, the poorer ability of smaller fish to osmoregulate in cold water has been argued as a reason for these non-spawning return migrations [95]. However, our results suggest that this could also be a response to an unfavorable P/G ratio caused by low winter growth. We predict unfavorable P/G ratios for the migratory environment when temperatures decrease in autumn and observe non-mature fish returning to rivers in our system. As our model does not explicitly include a change in size during the year, it may be overpredicting the frequency of such non-spawning migrants. Nevertheless, it appears likely that a small fish would not grow enough in one season to obtain a size-refuge from predators and will therefore be predicted to return by our model. To our knowledge, ours is also the first study that documents such non-spawning salmonid migrations in freshwater systems. Non-spawning salmonid migration is generally less researched than outmigration and spawning migration. As such, closer study of the mechanisms that drive these migrations can help us better understand migratory systems in general and may be a promising direction for future research.

## Conclusions

Our study provides strong conceptual support for predation risk versus growth tradeoffs as a determining factor for differential salmonid migration. Our modeling predictions of earlier migration of larger bodied individuals with lower predation risk in the feeding habitat are consistent with the empirically observed migratory timing. We conclude that increasing temperatures in spring create a better environment for growth, thereby influencing the trade-off between growth and predation which results in a temporally changing size-threshold that allows larger, harder-to-predate on fish to take advantage of the opportunities of early migration [55], while smaller fish migrate later in the season as growth opportunities increase further and start to outweigh the risk of mortality. These results may explain why a broad range of sizes, rather than a fixed threshold size for migration, is generally found in differential migration [14, 96, 97]. We suggest that similar tradeoffs could be an understudied factor of major importance in other systems, and a careful combination of theoretical modeling and empirical work in appropriate model systems may help to disentangle the extent of such effects. Furthermore, closer investigation of growth, mortality and their interactions may improve our understanding of differential migration systems.

## Supplementary information

**Additional file 1: Table S1.** Streams studied with coordinates of PIT-antennas and their distance over water to the lake. **Table S2.** Growth rate for residents and migrants based on recapture data. Migratory growth is significantly higher (*p* < 0.001 based on Type I SS Anova). **Table S3.** Number of tagged fish and migrants by stream. **Table S4.** Immature lake trout captured during fall spawning fisheries.

## Data Availability

All data collected during the course of this study will be archived in Dryad for public access and can be obtained from the corresponding author upon reasonable request. The code used to generate the model presented in this study is available from https://github.com/melian009/Migra.

## Abbreviations

F_ST_: The fixation index is an F-statistic that measures neutral genetic divergence between populations
G: Specific growth
M/M’: Individuals of a given size (M – small, M’ – large)
MS-222: Tricaine Methanosulfate, a muscle relaxant with anesthetic and analgesic properties specifically authorized for use in fish
P: Predation risk
P/G: An adaptation of the μ/G-ratio, where it is assumed that the main mortality risk is posed by predation
PIT-tags: Passive integrated transponder tags which are surgically implanted in fish and carry a unique identifying number that can be read by wire loop antennas
*P*_*Lmax*_ (*P*_*Smax*_): The carrying capacity for predators in a given system (L – lake, S – stream)
*α*_*L*_ (*α*_*S*_): The percentage of predators capable of preying on an individual of a certain size in the lake (L) and stream (S)
Δ*G(M)*: Growth differential between two habitats with unequal resource availability for individuals of a given size
μ/G: Mortality over growth, describing the tradeoff between the risk of mortality and the potential for growth. Low ratios, independent of actual values of μ and G, are considered favorable as the potential for growth outweighs the risk of mortality
