# Supplementary material for: Size-dependent tradeoffs in seasonal freshwater environments facilitate differential salmonid migration
Source: Mov Ecol. 2019 Dec 21;7:40. doi: 10.1186/s40462-019-0185-1 (PMC6925424; doi:10.1186/s40462-019-0185-1)
Supplement: Supplementary file 1 — Additional file 1: Table S1. Streams studied with coordinates of PIT-antennas and their distance over water to the lake. Table S2. Growth rate for residents and migrants based on recapture data. Migratory growth is significantly higher (p < 0.001 based on Type I SS Anova). Table S3. Number of tagged fish and migrants by stream. Table S4. Immature lake trout captured during fall spawning fisheries. [file 40462_2019_185_MOESM1_ESM.docx]

# Additional file

The full code used in producing the modeling results can be found on <https://github.com/melian009/Migra>.

Table S1: Streams studied with coordinates of PIT-antennas and their distance over water to the lake.
^1)^Gangbach and Walenbrunnen flow together to form Stille Reuss and are monitored by one antenna.

| Stream | Antenna coordinates | Distance antenna to lake (meters) |
| --- | --- | --- |
| Dorfbach LU | 47.012 N, 8.312 E | 189 m |
| Dorfach UR | 46.899 N, 8.622 E | 71 m |
| Gangbach^1)^ | 46.866 N, 8.632 E | 3824 m |
| Giessen | 46.898 N, 8.619 E | 52 m |
| Klosterbach UR | 46.899 N, 8.605 E | 101 m |
| Leewasser | 46.995 N, 8.605 E | 363 m |
| Mühlibach | 46.980 N, 8.338 E | 50 m |
| Polenschachen | 46.804 N, 8.660 E | 11720 m |
| Scheidgraben | 46.982 N, 8.418 E | 9 m |
| Schützenbrunnen | 46.810 N, 8.662 E | 11170 m |
| Stille Reuss^1)^ | 46.866 N, 8.632 E | 3824 m |
| Walenbrunnen^1)^ | 46.866 N, 8.632 E | 3824 m |

Table S2: Growth rate for residents and migrants based on recapture data. Migratory growth is significantly higher (p < 0.001 based on Type I SS Anova).

| Migratory Strategy | No. of individuals | Average daily growth ± (mm) |
| --- | --- | --- |
| Resident | 279 | 0.17 ± 0.12 |
| Migratory | 30 | 0.41 ± 0.12 |

Table S3: Number of tagged fish and migrants by stream

| Stream | Fish tagged | Migrants |
| --- | --- | --- |
| Dorfbach LU | 179 | 19 |
| Dorfbach UR | 246 | 60 |
| Gangbach | 317 | 31 |
| Giessen | 288 | 121 |
| Klosterbach UR | 359 | 174 |
| Leewasser | 331 | 17 |
| Mühlibach | 458 | 31 |
| Polenschachen | 132 | 42 |
| Scheidgraben | 401 | 96 |
| Schützenbrunnen | 404 | 110 |
| Stille Reuss | 356 | 61 |
| Walenbrunnen | 341 | 62 |

Table S4: Immature lake trout captured during fall spawning fisheries

| River | Date | Total length (mm) | Weight (g) |
| --- | --- | --- | --- |
| Giessen | 17.11.2015 | 225 | 99.70 |
| Giessen | 13.11.2017 | 313 | 265.10 |
| Klosterbach UR | 15.11.2016 | 202 | 61.80 |
| Klosterbach UR | 13.12.2016 | 306 | 276.60 |
| Mühlibach | 24.11.2017 | 250 | 142.80 |
| Mühlibach | 24.11.2017 | 324 | 380.00 |
| Mühlibach | 07.12.2017 | 283 | 204.50 |
| Mühlibach | 07.12.2017 | 354 | 321.50 |
| Scheidgraben | 06.12.2016 | 289 | 241.30 |
| Scheidgraben | 06.12.2016 | 262 | 181.30 |
